# Observation of Electroplating in a Lithium-Metal Battery Model Using Magnetic Resonance Microscopy

**DOI:** 10.3390/molecules30132733

**Published:** 2025-06-25

**Authors:** Rok Peklar, Urša Mikac, Igor Serša

**Affiliations:** 1 Jožef Stefan Institute, 1000 Ljubljana, Slovenia; rok.peklar@ijs.si (R.P.); ursa.mikac@ijs.si (U.M.); 2 Jožef Stefan International Postgraduate School, 1000 Ljubljana, Slovenia; 3 Institute of Anatomy, Faculty of Medicine, University of Ljubljana, 1000 Ljubljana, Slovenia

**Keywords:** lithium-metal batteries, dendritic growth, structure analysis, charging regimes, symmetric cell, MRI

## Abstract

Accurate imaging methods are important for understanding electrodeposition phenomena in metal batteries. Among the suitable imaging methods for this task is magnetic resonance imaging (MRI), which is a very powerful radiological diagnostic method. In this study, MR microscopy was used to image electroplating in a lithium symmetric cell, which was used as a model for a lithium-metal battery. Lithium electrodeposition in this cell was studied by sequential 3D ^1^H MRI of 1 M LiPF_6_ in EC/DMC electrolyte under different charging conditions, which resulted in different dynamics of the amount of electroplated lithium and its structure. The acquired images depicted the electrolyte distribution, so that the images of deposited lithium that did not give a detectable signal corresponded to the negatives of these images. With this indirect MRI, phenomena such as the transition from a mossy to a dendritic structure at Sand’s time, the growth of whiskers, the growth of dendrites with arborescent structure, the formation of dead lithium, and the formation of gas due to electrolyte decomposition were observed. In addition, the effect of charge and discharge cycles on electrodeposition was also studied. It was found that it is difficult to correctly predict the occurrence of these phenomena based on charging conditions alone, as seemingly identical conditions resulted in different results.

## 1. Introduction

Rechargeable lithium-metal (Li-metal) batteries are considered one of the most promising energy storage technologies, primarily due to lithium’s extremely low redox potential, high theoretical specific capacity, and low density [1,2,3]. Research into Li-metal anodes dates back to the 1970s [4], yet their widespread commercial application has been hindered by the persistent challenge of lithium dendrite formation during charging. These dendrites can pierce the separator, leading to internal short circuits and posing serious risks such as thermal runaway [2,3]. Dendrite formation arises from several factors, including uneven anode surfaces, the instability of the solid electrolyte interphase (SEI), electrolyte decomposition, and high local current densities. Furthermore, repeated charge–discharge cycling can lead to the formation of electrically isolated “dead” lithium, i.e., metallic lithium that becomes disconnected from the current collector during stripping. This not only reduces the pool of active lithium but also degrades the SEI, accelerating electrolyte decomposition and diminishing Coulombic efficiency, ultimately shortening battery life.

Despite extensive research on the subject, the mechanisms governing lithium deposition remain only partially understood. Various models have been proposed to elucidate this process and identify the factors responsible for nonuniform deposition [5]. One widely accepted model is the space-charge model [6,7], which delineates two regimes based on the applied current density. Below the limiting current density (*j* < *j_lim_*), lithium growth is reaction-limited. In this regime, the concentration gradient in the electrolyte reaches a steady state, resulting in a stable ion distribution that promotes uniform Li deposition. However, at higher current densities (*j* > *j_lim_*), ion transport becomes diffusion-limited, resulting in ion depletion near the negative electrode surface. Eventually, the ion concentration at the electrode surface drops to zero at Sand’s time, beyond which dendritic growth initiates and grows at a constant velocity. Based on the model’s findings, strategies such as lowering the local current density, increasing the initial salt concentration, or enhancing the cation transference number have been proposed to extend Sand’s time. However, other models have shown that nonuniform Li deposition can also occur at current densities below *j_lim_* and/or at times shorter than Sand’s time, i.e., in the reaction-limited regime. In this regime, lithium may deposit in mossy form or as metallic filaments (e.g., whiskers or needles), with the morphology strongly influenced by the SEI’s composition and structure [8]. Whisker growth, in particular, has been linked to mechanical stresses beneath the SEI that cause it to fracture, creating pathways for lithium to protrude [9]. Phase-field models have offered further insights into the detailed structures of deposited lithium [10,11]. These models show that at low voltages, lithium grows in fibre-like forms, while higher voltages first trigger fully dendritic, then tip-splitting dendritic growth patterns. The onset of these morphological transitions is highly sensitive to the size of surface protrusions, with larger ones lowering the voltage threshold for dendrite formation. Additionally, pulse charging has been shown to suppress dendritic growth even at higher voltages [12], a conclusion supported by film growth models [13], which also suggest that engineering 3D, lithiophilic anode substrates can promote smoother, more uniform lithium deposition. To predict the macroscopic evolution of dendritic structures, a diffusion-limited aggregation model incorporating both diffusion and electromigration, reflecting the conditions during lithium electrodeposition, was developed. It revealed that combined effects induce anisotropic dendritic growth, with structures preferentially propagating along the electric field [14]. Different models thus show that factors such as applied voltage, temperature, electrolyte composition, electrode surface roughness, and the presence of impurities play significant roles in the SEI composition and, consequently, in lithium deposition [8,10,11,15,16].

Another critical challenge during battery cycling is the irreversible loss of active lithium. Computational modelling has demonstrated that the formation of dead lithium leads to a pronounced increase in internal resistance and capacity fade. Consequently, the occurrence of dead lithium is accompanied by a rise in cell voltage, making voltage monitoring an effective method for detecting its accumulation during cycling [17].

To validate model predictions and gain deeper insight into the complex mechanisms of lithium dendrite growth, in situ characterization techniques have been developed [2]. These provide real-time insights into dendrite formation, each with distinct compromises in spatial and temporal resolution and compatibility with realistic battery architectures. Experimental studies have confirmed the presence of various lithium structures—mossy, whisker-like, needle-shaped, tree-like, and bushy—under different electrochemical conditions, consistent with the predictions of the theoretical models [8,15,18,19,20,21,22,23,24]. These morphologies are strongly influenced by current density and charging time, which is consistent with the predictions of the space-charge model [15,18,20,21,22,25]. Root-like lithium growth typically occurs under reaction-limited conditions, influenced by SEI properties and electrode volume changes [8]. At higher applied voltages, the SEI forms faster, which promotes whisker growth through cracking, whereas a stable, slowly formed SEI favours mossy deposition. As the reaction-limited regime shifts to a diffusion-limited regime, lithium grows dendritically, coinciding with a sharp voltage rise [21]. Further supporting the space-charge model, dendritic growth is often observed in regions of electrolyte ion depletion [15,19,25]. However, experiments also show that dendrites can form at lower current densities or earlier than predicted by Sand’s time—which was attributed to local ion depletion around surface protrusions elevating local current densities and effectively shortening Sand’s time [22,25,26,27]. Moreover, the coexistence of two distinct types of deposited lithium has been observed: Type I, which is thicker and composed of partially oxidized lithium, and Type II, which is thinner, composed of brittle lithium hydride (LiH), and more readily detaches and contributes to dead lithium. Since LiH forms through reactions with hydrogen, hydrogen-free electrolytes can suppress its formation, thereby improving battery life [16]. The formation of electrically isolated dead lithium has also been experimentally observed [8]. Additionally, applying mechanical pressure during cycling densifies lithium deposits, suppresses dendrite growth, enhances Coulombic efficiency, and extends cycle life [28]. To address these challenges and promote uniform mossy lithium deposition, various strategies have emerged, including electrolyte formulation [29,30], the structural design of Li-metal anodes [31,32], the minimization of lithium anode volume expansion during cycling [33], SEI engineering [34,35], the development of advanced separators, the application of external pressure [28], and the optimization of cycling protocols [36].

This study explores the application of 3D ^1^H MRI to monitor the evolution of different lithium-deposit morphologies during battery operation. Despite limitations in spatial and temporal resolution, along with susceptibility artifacts and eddy current effects, magnetic resonance offers a non-invasive, multifaceted approach to studying lithium deposition mechanisms. However, successful imaging requires the use of non-magnetic components and precise alignment of metallic parts to minimize artifacts [37,38]. It was shown before that ^7^Li NMR and MRI enable the differentiation of lithium morphologies (e.g., metallic, mossy, dendritic) and quantification of salt diffusivity and ion transference numbers [25,39,40,41,42,43,44] and that dual ^7^Li/^19^F MRI allows simultaneous mapping of cation and anion distributions in fluorinated electrolytes [39]. A major challenge with ^7^Li MRI is its inherently weak signal compared to that of ^1^H, which limits the ability to perform fast, high-resolution imaging of dendritic structures. However, since most electrolytes contain protons, ^1^H MRI can indirectly visualize lithium deposition by detecting voids in the electrolyte, enabling high-resolution 3D images [23,24]. Our study demonstrates that ^1^H MRI enables real-time visualization of lithium morphology evolution in symmetric Li-metal cells under varying charging conditions.

## 2. Results

The following subsections present various phenomena observed by MR microscopy during lithium plating in a lithium symmetric cell, used as a model for a lithium-metal battery. The cell was filled with a 1 M LiPF_6_ (EC:DMC = 1:1) electrolyte that produced a detectable MR signal and appeared bright on the MR images. In contrast, the lithium electrodes and structures upon them appeared dark in MR images, as they did not produce any MR signal. As shown previously, lithium deposits can also contain hydrogen in the form of lithium hydride [16]. However, since the deposits are solid, lithium hydride does not produce a detectable ^1^H MRI signal due to its short spin–spin relaxation time *T*_2_. In addition, dark areas in the MR images may also result from bubbles formed by electrolyte decomposition.

The cell design was optimized for MR microscopy, meaning that the cells did not contain any metal parts other than electrodes and current collectors, and their orientation with respect to the static magnetic field *B*_0_ and the radiofrequency field *B*_1_ was chosen so that the effects of magnetic susceptibility and eddy currents on the MR image were minimized [37,38]. Three-dimensional MR images were recorded to observe all deposited lithium and to determine shapes and positions of the deposits on the anode during battery charging. Additionally, the 3D images help distinguish between deposited lithium and the bubbles formed by electrolyte decomposition based on their 3D shapes—bubbles are spherical, whereas dendrites are expected to have different morphologies. In the following subsections, mostly representative slices (2D images) are shown.

### 2.1. Sand’s Time

The Sand’s time corresponds to the time when the concentration of ions in the electrolyte at the electrode surface drops to zero and consequently dendritic growth is initiated. It is given by the equation [21,45]

(1) $$t_{S}=\frac{\pi D_{+}{\left(zFc_{0}\right)}^{2}}{4{\left(jt_{a}\right)}^{2}}$$

where $D_{+}$ is the diffusion constant of the Li cation, which was taken as equal to 2.3 × 10^−10^ m^2^/s (at room temperature), *z* is the charge number and is equal to 1 for Li^+^, *F* is Faraday’s constant, $c_{0}$ is the initial Li^+^ concentration in the electrolyte, *j* is the current density, and $t_{a}$ is the transference number between the Li cation and the corresponding anions, which was taken as equal to 1 − 0.3 = 0.7. For the Li symmetric cell setup in this study, the corresponding Sand’s time as a function of current density is given in Table 1.

### 2.2. Transition from Mossy to Dendritic Structure

**Figure 1:**
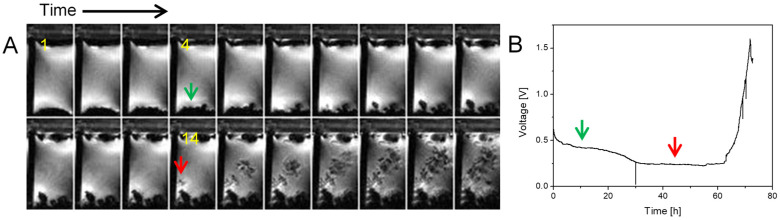
Time-lapse MR images of a representative cross section through a symmetrical lithium cell (**A**) and the corresponding time dependence of the supply voltage (**B**). A constant current density of 1 mA/cm^2^ was used to charge the cell. The cathode is at the top and the anode at the bottom of the images, and the frames are 200 min apart. Lithium plating has a mossy structure up to frame 14 (45 h) and then transitions to a dendritic one. The red arrow points to the onset of first dendrites, while the green one points to the mossy structure in frame 4 where the onset of first dendrites was expected according to the Sand’s time.

Previous studies have observed transitions of initially mossy structures to different dendritic morphologies, such as needle-like [20], finger-like [21], or microstructure forms [22]. This transition may result from a shift from a reaction-limited to a diffusion-limited process [2,15,21], which, according to theory, should start at Sand’s time. In the example shown in Figure 1, the applied current density through the cell was 1 mA/cm^2^ and the corresponding Sand’s time was 9.6 h (Table 1). Since the time difference between successive frames is 200 min, the first dendrites should be observable in the fourth frame. However, dendrites are not observed until the 14th frame. This initiation of dendrite growth was not accompanied with a jump in the supplied voltage, as was expected [21]. The reason for this may be the concentration gradient of Li^+^ and the resulting uneven distribution of current density [22]. After the transition from a mossy to dendritic structure, the rate of Li^+^ deposition accelerated significantly.

**Figure 2:**
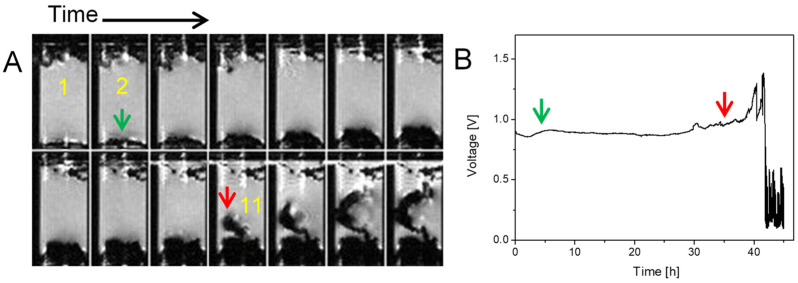
Time-lapse MR images of a representative cross section through a symmetrical lithium cell (**A**) and the corresponding time dependence of the supply voltage (**B**). A constant current density of 1.5 mA/cm^2^ was used to charge the cell and frames are 200 min apart. The cathode at the top (the anode is the bottom) also has some lithium dendrites because the cell was charged with a countercurrent for a day before starting this MRI experiment. Lithium plating has a mossy structure up to frame 11 (35 h), then it transitions to a dendritic structure. The red arrow points to the onset of first dendrites, while the green one in frame 2 denotes the time point when the onset of first dendrites was expected according to the Sand’s time.

In the second case, shown in Figure 2, the same type of symmetric lithium cell was used but the charging current density was increased to 1.5 mA/cm^2^. This cell was charged for one day before insertion into the MR magnet and sequential imaging began, with charging continuing in the reverse direction of the current. In this experiment, the lithium plating changed from a mossy structure to a different type of dendrite than in the previous case (Figure 1). Here, the structure still looks as dense, but as can be seen in frame 11 (red arrow) and later frames, rapid growth in the form of a thin dendrite took place. This shape could be explained by cracks in the SEI that appeared at this time, so that a thin dendrite like a whisker started to grow from the crack. This dendrite does not have a constant diameter and branches in the last frame [8]. Again, the first dendrites were not observed at Sand’s time, i.e., at 4.2 h, but at frame 11, which corresponds to 35 h. The increase in voltage was observed after frame 12 (at 38 h).

### 2.3. Simultaneous Presence of Small Dense and Thin Filamentous Dendrites

**Figure 3:**
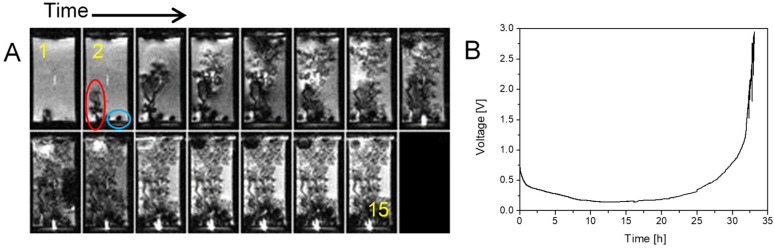
Time-lapse MR images of a representative cross section through a symmetrical lithium cell (**A**) and the corresponding time dependence of the supply voltage (**B**). A constant current density of 1.5 mA/cm^2^ was used to for charging the cell and frames are 200 min apart. The anode is at the bottom. In the MR images can be seen the coexistence of small, dense, slow-growing dendrites (blue bordered) and thin, filamentous, fast-growing (red bordered) dendrites.

The following example in Figure 3 presents a case where two distinctly different types of dendrites are simultaneously present, namely small, dense, and slow-growing dendrites and thin, filamentous, and fast-growing dendrites. The conditions in the cell and the experiment were the same as those in the previous experiment in Figure 2 (current density of 1.5 mA/cm^2^, Sand’s time of 4.2 h), except that the current direction was constant this time. Due to the high current density, a diffusion-limited regime was present from the second frame onwards. This could also explain the early coexistence of these two different types of dendrites. Perhaps both types existed already towards the end of the reaction-limited regime. The coexistence of two types of deposited lithium have been also observed by the cryo-STEM method that additionally revealed that these two different types of dendrites also have different compositions; the small, dense dendrites are composed of partially oxidized lithium (blue bordered), while the thinner, filamentous dendrites are composed of lithium hydride (red bordered) [16]. During this experiment, a significant amount of electrolyte was expelled out of the cell by the pressure of gases generated from the decomposed electrolyte. This resulted in an increase in the current density in the remaining electrolyte-filled part of the cell and in the voltage.

### 2.4. Current Cycling and the Formation and Breakdown of Whiskers

**Figure 4:**
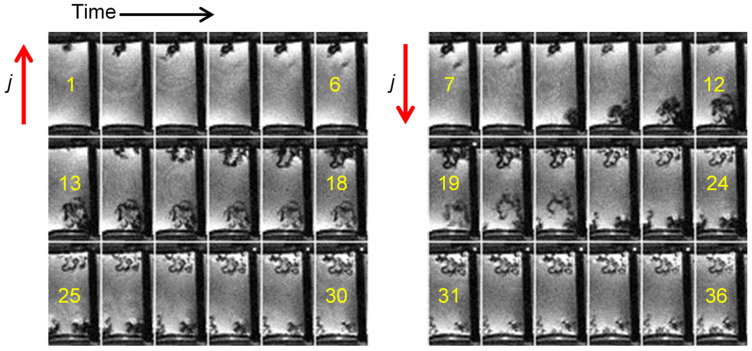
Time-lapse MR images of a representative cross section through a symmetrical lithium cell. The cell was charged and then discharged (by reversing the current) every six frames (20 h). The charging regime (*j* = 0.5 mA/cm^2^, Sand’s time of 38.2 h) was such that the experiment was performed in the reaction-limited regime. The experimental conditions were favourable for the growth of lithium whiskers, which is clearly visible from frame 10 onwards. In frames 1–6, the anode is the top electrode and dendrites are formed there first. When the current is reversed (in frames 7–12), the dendrites in the top electrode begin to decompose and almost disappear by frame 12, while they start to grow on the new anode (bottom electrode). A similar effect, but to a lesser extent, is also visible in later frames with each change in the direction of the current.

The following experiment in Figure 4 shows an example where a symmetric lithium cell was subjected to charge and discharge cycles, such that the current direction was reversed every six frames (every 20 h). Furthermore, the current flowed for only one hour between two consecutive frames, i.e., a total of six hours in a 20 h interval. Since the current density was equal to 0.5 mA/cm^2^ and the Sand’s time was 38.2 h, this experiment was well performed in the reaction-limited regime. The result of these conditions was the growth of whiskers or hairy dendrites with a constant diameter and a twisted structure. The structure of the resulting dendrites in the reaction-limited regime depends on the electric potential, temperature, electrolyte composition, electrode surface, and the SEI [8,25]. In case of high overpotential, long and twisted lithium hairs (lithium whiskers) are often formed. In Figure 4, the effect of the cyclical current direction change is also clearly visible. In frames 1–6, dendrites first grow on the top electrode; when the current is reversed, these dendrites shrink and virtually disappear, while dendrites (whiskers) grow on the bottom electrode. Dead lithium was also observed in the other slice, as predicted by the phase field model describing the lithium stripping process [17]. With further cycling, the reduction of dendrites (whiskers) due to the reversed current becomes less effective.

### 2.5. Dendrites with Arborescent Structure

**Figure 5:**
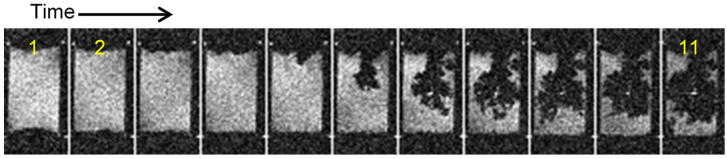
Time-lapse MR images of a representative cross section through a symmetrical lithium cell. A constant current density of 1.6 mA/cm^2^ was used to charge the cell, the frames are 200 min apart, and the anode is at the top. The MR images show that the emerging dendrites have an arborescent structure.

In the following example in Figure 5, the current density was equal to 1.6 mA/cm^2^, so the Sand’s time was 3.7 h—somewhat shorter than in previous experiments. At such high currents (in the Sand’s regime), dendrites grow due to the concentration gradient of Li^+^ ions. Along the electrode, dendrites can grow at different rates due to the inhomogeneity of the current density, which is a consequence of local defects [15,18,19,25]. In this experiment the obtained dendrites had an arborescent structure.

### 2.6. Dead Lithium

**Figure 6:**
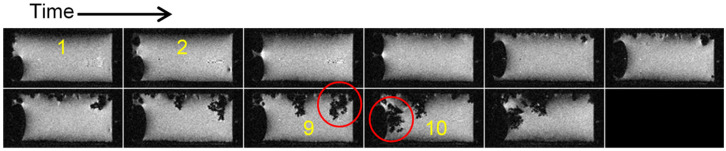
Time-lapse MR images of a representative cross section through a symmetrical lithium cell. The cell was charged at a current density of 2.5 mA/cm^2^, the anode is at the top, the electrolyte surface is on the left, and the frames are 200 min apart. A red-bordered dendrite in frame 9 is still attached to the anode, while in frame 10 it has already broken off and migrated to the electrolyte surface.

There may be forces present in the liquid electrolyte that can tear off part of the lithium dendrite from the electrode [8]. This lithium then moves towards the surface due to buoyancy, since lithium is less dense than the electrolyte. Forces that, in addition to buoyancy, can first cause the lithium dendrite to break off and then migrate with a microcurrent are convection due to heating, electroosmosis, electrocapillary forces, etc. Such torn off lithium can be considered dead in an electrochemical sense, since it cannot contribute to the battery capacity. Instead, it is more likely that it can cause a short circuit in the battery. An example of a torn off dendrite is shown in Figure 6. In frame 9, the red-bordered dendrite is still part of the anode, then it breaks off and in frame 10 and has already moved on the surface of the electrolyte.

### 2.7. Gas Bubbles

**Figure 7:**
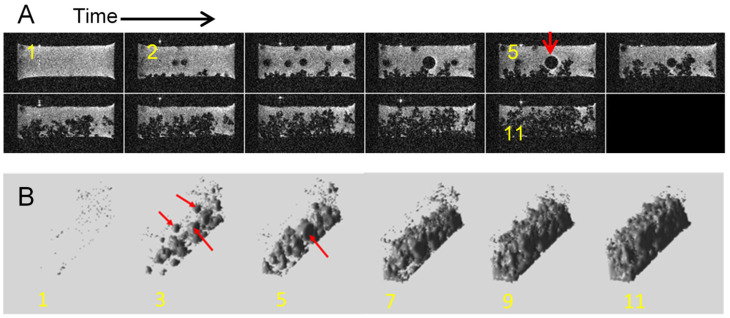
Time-lapse MR images of a representative cross section through a symmetrical lithium cell (**A**) and a 3D reconstruction of signal voids, obtained via threshold filtering (**B**). The cell was charged at a current density of 1.6 mA/cm^2^, the anode is at the bottom, and the frames are 200 min apart. The red arrow in frame 5 points to a gas bubble. Several other bubbles can be seen in other frames.

In cases where the charging parameters exceed the tolerances of the electrolyte, it may begin to decompose [16]. This process is accompanied by the formation of gases. Gas in the electrolyte can be seen as round bubbles. These can remain attached to the electrodes or the cell walls due to surface tension forces. However, the bubbles can also move to the surface of the electrolyte. In MR images, gas bubbles can be seen as signal voids, which are relatively easy to distinguish from dendrites due to their characteristic round shape. Figure 7 shows an example of a cell where gas bubbles were formed during charging. Panel (A) shows time lapse images of a selected representative slice across the cell, and panel (B) shows the corresponding volume rendered images of signal voids.

**Figure 8:**
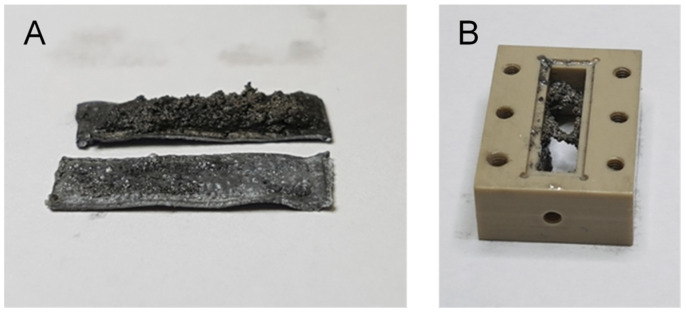
Photographs of the electrodes (**A**) and the central block of the cell (**B**) taken immediately after the cell was disassembled in the experiment shown in Figure 7. The photographs show the complex dendritic structure that formed in the anode ((**A**), top electrode) of the battery during charging. Since this structure is very fragile, it broke during disassembly and a significant part of it remained in the central block of the cell. The cathode ((**A**), bottom electrode) remained practically flat.

The cell of this experiment was disassembled after the experiment was completed in order to visually inspect the dendrites that were formed during charging. The disassembled cell is shown in Figure 8, with photographs of both electrodes (A) and the central block of the cell (B). It can be seen that large dendrites had grown on the anode, which broke during disassembly, leaving only a part on the anode (the upper electrode in Figure 8A) and the other part in the central block (Figure 8B). The cathode (the lower electrode in Figure 8A) remained practically flat compared to the anode.

## 3. Discussion

The aim of this study is to demonstrate that ^1^H MR microscopy of electrolyte distribution can provide useful insights into important phenomena in metal batteries. Accurate nondestructive imaging methods are important in battery research, especially in metal batteries, where electrodeposition processes are often not yet fully understood. In this study, the focus was on lithium-metal batteries, but other metal batteries could also be studied with this method [46]. Although our main purpose was to observe the growth of dendrites on the anode, we sometimes unexpectedly found other structures, e.g., whiskers in Figure 4. Gas bubbles also show up as ellipsoidal/spherical signal voids. The formation of CO_2_, C_2_H_4_, and trace H_2_ is attributed to reductive decomposition of the carbonate solvents [47]. PEEK is generally inert to carbonate solvents, while the photopolymerization resin safety data sheet lists no reactivity with carbonates. The chemical stability of the resin was tested by monitoring the cell using MRI under open-circuit conditions for 24 h, with no detected bubbles or significant change in mass.

Transitions between different structures were not always found exactly when theoretically expected, e.g., the transition from mossy to dendritic structure in Figure 2 and Figure 3. The onset of this transition is determined by Sand’s time, when the local concentration of Li^+^ ions in the electrolyte at the anode is depleted, so that electrodeposition transits from a reaction-limited to a diffusion-limited regime. In our study, the theoretical values predicted earlier effects than actually occurred in the experiment. During the initial phase of electrodeposition, part of the total current is diverted to the growth of the SEI and only the plating current depletes cations. Modified “Sand” models include lithium transport across the SEI and predict a longer onset time [48]. According to the modified model, the dendrite onset time depends on the physiochemical and transport properties of the SEI and is inversely proportional to the SEI growth rate, which is slower in cells with a larger distance between the electrodes [49]. Therefore, in our experiments, where the distance between the electrodes was large (8 mm or 10 mm), the onset of the diffusion-limited regime is expected to occur at longer times, as predicted by Sand’s time (Equation (1)). A major effect of a large, separator-free gap compared to a thin porous separator (~50 μm) is that natural convection rolls are much better supported, essentially smoothing out Li ion concentration gradients and leading to a longer time before transition into a diffusion-limited regime [50]. Related work in the field of acoustic streaming showed that even a very weak flow can increase the limiting current by an order of magnitude [51]. While the onset of transition is found to always be at later times than estimated by the Sand equation, the observed delays display a stochastic nature; current-density hot spots, and the buoyancy-driven convection cells that sprout from those sites, are inherently different in each cell, resulting in a formation of a unique flow/field pattern [52]. Other interesting phenomena observed with ^1^H MR microscopy of electrolyte distribution were the formation of dead lithium and the decomposition of the electrolyte. Both phenomena can significantly reduce battery performance and dead lithium poses a serious safety issue.

The ability to detect all these effects can be attributed to the noninvasiveness of MRI as a detection method. An advantage of MRI is its ability to image the electrolyte directly, provided that it is a liquid and that it contains hydrogen atoms, while the disadvantages of MRI are its relative slowness and moderate spatial resolution. Another challenge is the minimization of artifacts due to metallic parts [37], which also all need to be nonmagnetic. With the method used it was not possible to detect lithium structures directly, only indirectly as signal voids. The relationship between voxel fill fraction and signal attenuation is highly non-linear, with small volumes of deposited lithium causing disproportionately large signal loss relative to their size. The amount of signal loss is determined mainly by *B*_0_ and *B*_1_ perturbations in the vicinity of metallic lithium and is dependent on the shape and orientation of lithium deposits relative to *B*_0_ and *B*_1_. This, together with the point-spread function inherent to MRI, means that signal void maps will always overestimate the true volume of deposited lithium [24]. A direct lithium detection is possible by ^7^Li NMR/MRI, but this experiment is very challenging due to poor SNR [41]. Furthermore, because the paramagnetic Li metal is subject to strong susceptibility gradients, Knight-shift broadening, and RF skin-depth effects, it suffers severe distortion and signal loss compared to indirect ^1^H imaging [24,53]. The hardware for ^1^H imaging is readily available, making another case for imaging the electrolyte instead of the Li metal. A hybrid approach presents itself; the sequential use of a double-tuned coil or two nested coils would allow the use of ^1^H imaging for faster, high-resolution scans, while occasional ^7^Li scans would allow mapping of the distribution of lithium-metal and lithium-ion concentrations in the electrolyte directly, as well as enable chemical shift imaging (CSI), which was demonstrated to differentiate between dendritic and bulk metal in situ, as well as various lithium salts [41,54].

There are other imaging methods that could be used for this purpose, but they all have their own specificities, advantages, and disadvantages. Comparable or better resolution imaging can be obtained using µCT. This method has also an advantage in comparison to MRI in that it is faster and not sensitive to the presence of metallic parts; in fact, these images give most of the contrast that is available in CT images. However, µCT cannot image the electrolyte well and formations like bubbles cannot be detected. A much better resolution can be obtained via SEM [16], although this method requires specific cell design and operation under a high-vacuum environment. This makes it difficult to dynamically observe the electroplating progress and limits the choice of electrolyte. Probably the simplest method for the observation of electroplating is via photographing a transparent cell [20,21]. Such a cell can be made, for example, from plexiglass using a similar design to that used in this study. This kind of cell allows a side view of the anode, which can be efficient in early stages of electroplating when the dendrites are still small, while later it would be practically impossible to resolve the spatial distribution of dendrites or other more complex structures between the electrodes.

The lithium symmetric cell used in this study was optimized for MRI and therefore had a much larger electrode spacing than is used in real batteries. This enabled electrodeposition of much larger structures than are found in practice. However, the phenomena detected with these model batteries are important and also occur in real batteries. Another peculiarity is due to the indirect imaging of electrodeposition. All metal structures detected with this method appeared larger in the images than they actually were. This is because the signal void is not limited to the actual space occupied by the metal structure but can be much larger due to the effects of eddy currents induced in the metal structures due to signal attenuation [23,24]. To reduce these artifacts, spin-echo-based MRI sequences, which are less susceptible to *B*_0_ inhomogeneities, should be used. In addition, RF and static magnetic field modifications induced by Li metal can be calculated and used in MR images to more accurately quantify the amount of Li dendrites [24].

## 4. Materials and Methods

### 4.1. Lithium Symmetric Cell

**Figure 9:**
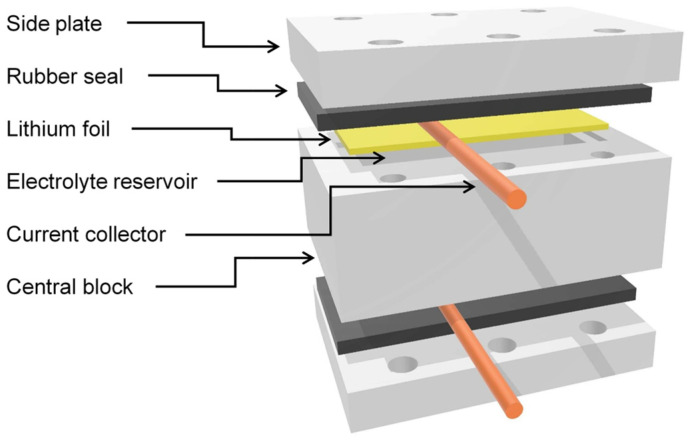
Schematic of the symmetric cell. The cell design was optimized for MR imaging.

Lithium dendrite growth was investigated in lithium symmetric cells under different charging regimes. The cells were made in two slightly different sizes. The smaller one had electrodes measuring 16 × 4 mm^2^ at a distance of 8 mm, while the larger one had electrodes measuring 20 × 5 mm^2^ at a distance of 10 mm. The cell housing materials were manufactured either from PEEK or by 3D printing using stereolithography with Phrozen TR300 resin (Phrozen Tech Co., Ltd., Hsinchu City, Taiwan). Both electrodes (anode and cathode) were made of 0.38 mm thick lithium-metal foil, which was cut to dimensions 1 mm larger than the above-mentioned active size to fit into a recess in the central block of the cell housing and thus enable a good seal. The space between the electrodes was filled with an electrolyte of 1 M LiPF_6_ in a 1:1 volumetric mixture of ethylene carbonate (EC) and dimethyl carbonate (DMC). A thin strip of copper wire was laid across the electrodes on the outside, serving as a current collector, and a silicone rubber sheet and the outer side plate of the housing were placed on top. This sandwich of layers, which was identical on both sides of the cell, was then pressed to the central block of the housing with six nylon screws on each side of the cell. Once the cell was assembled, it was filled with electrolyte through a small hole in one side of the central block, and the hole was then sealed with a rubber O-ring and a nylon screw. A schematic representation of the cell, showing all the cell components with their size proportions and arrangement, is shown in Figure 9. The cell was assembled in a glove box (Vigor Gas Purification Technologies, Marktheidenfeld, Germany) under an inert argon atmosphere. All chemicals for the cell were purchased from Sigma Aldrich (Merck, Burlington, MA, USA). The housing components, the central block, and the two side plates were fabricated from peek plastic using a CNC machine.

### 4.2. Magnetic Resonance Imaging

Symmetric cells were imaged by sequential magnetic resonance imaging at high spatial resolution during their charging. Two different imaging sequences were used for imaging. The first was a standard three-dimensional spin-echo (SE) imaging sequence, and the second one was its faster version, the three-dimensional rapid acquisition with relaxation enhancement (RARE) method. The method parameters for different experiments of the study are shown in Table 2. A new image was acquired every 3 h and 20 min. During this interval, the cell was charged for one hour or continuously. In the first case, the charging conditions were set with a constant voltage of 3.2 V or 4.3 V between the electrodes, while in the second case a constant current of 1 mA or 1.5 mA was applied. In the latter case, the charging was monitored by a potentiostat (VSP, Bio-Logic, Claix, France), with which the *V*(*t*) curve was measured. The magnet bore (and consequently the cell) is thermostated at 20 °C by a water-chilled cooler for gradient cooling.

Imaging was performed on an MR system consisting of a 9.4 T (proton frequency 400 MHz) wide-bore vertical NMR magnet (Jastec, Tokyo, Japan), a fully digital NMR/MRI spectrometer (Redstone, Tecmag, Houston, TX, USA), and radiofrequency (RF) and gradient coils for MR microscopy (Micro 2.5, Bruker, Ettlingen, Germany). A 30 mm RF coil operating in linear mode was used for signal detection and RF excitation.

## 5. Conclusions

This study demonstrates that the complex electrodeposited structures that can occur in metal batteries during their operation can also be successfully investigated using indirect MRI. The study was performed on a lithium symmetric cell as a battery model, but the method used can also be performed with electrodes of other metals and even with real batteries, provided that the electrolyte contains hydrogen atoms with sufficiently long *T*_2_ relaxation times to still allow MR imaging and that the cell is optimized for MR signal reception.

## Figures and Tables

**Table 1:** Sand’s time as a function of current density of the Li symmetric cell setup of this study.

*j* [mA/cm^2^]	*t_S_* [h]
0.5	38.2
1	9.6
1.5	4.2
2.5	1.5

**Table 2:** Imaging parameters * of the presented images.

Figure	Imaging Sequence	FOV [mm^3^]	Matrix	TE/iTE [ms]	TR [ms]	NEX	RARE Factor
Figure 5, Figure 6 and Figure 7	3D SE	20 × 10 × 5	128 × 64 × 32	22	2000	1	/
Figure 4	3D SE	24 × 12 × 6	128 × 64 × 32	5	2000	2	/
Figure 1, Figure 2 and Figure 3	3D RARE	24 × 12 × 6	128 × 64 × 32	5.7	2000	8	4

* FOV—field of view, TE—echo time, iTE—inter echo time, TR—repetition time, NEX—number of excitations, RARE factor—number of *k*-space lines acquired in one signal excitation.

## Data Availability

The data presented in this study are available from the corresponding author upon request.

## Abbreviations

The following abbreviations are used in this manuscript:

CNC	Computer numerical control
DMC	Dimethyl carbonate
EC	Ethylene carbonate
MR	Magnetic resonance
MRI	Magnetic resonance imaging
MRM	Magnetic resonance microscopy
NMR	Nuclear magnetic resonance
RARE	Rapid acquisition with relaxation enhancement
SE	Spin echo
SEI	Solid electrolyte interphase
SEM	Scanning electron microscopy
TEM	Transmission electron microscopy
µCT	Computed tomography microscopy
